# A synoptic account of flora in the National Wetland Park of the Alpine Permafrost Zone, Jimunai, Xinjiang

**DOI:** 10.3897/BDJ.13.e160471

**Published:** 2025-12-22

**Authors:** Qiumei Cao, Xiaoling Fan, WenJun Li, Bin Liu, Zheping Xu

**Affiliations:** 1 Grassland College, Xinjiang Agricultural University, Urumqi, China Grassland College, Xinjiang Agricultural University Urumqi China; 2 State Key Laboratory of Ecological Safety and Sustainable Development in Arid Lands, Xinjiang Institute of Ecology and Geography, Chinese Academy of Sciences, Urumqi, China State Key Laboratory of Ecological Safety and Sustainable Development in Arid Lands, Xinjiang Institute of Ecology and Geography, Chinese Academy of Sciences Urumqi China; 3 Xinjiang Key Laboratory of Biodiversity Conservation and Application in Arid Lands, Xinjiang Institute of Ecology and Geography, Chinese Academy of Sciences, Urumqi, China Xinjiang Key Laboratory of Biodiversity Conservation and Application in Arid Lands, Xinjiang Institute of Ecology and Geography, Chinese Academy of Sciences Urumqi China; 4 Xinjiang Normal University, Urumqi, China Xinjiang Normal University Urumqi China; 5 National Science Library, Chinese Academy of Sciences, Beijing, China National Science Library, Chinese Academy of Sciences Beijing China; 6 Department of Information Resources Management, School of Economics and Management, University of Chinese Academy of Sciences, Beijing, China Department of Information Resources Management, School of Economics and Management, University of Chinese Academy of Sciences Beijing China

**Keywords:** Alpine permafrost zone, Jimunai National Wetland Park, plant diversity, endemic species, conservation status, new records

## Abstract

**Background:**

The alpine periglacial wetland, situated near the alpine snow belt, represents one of the most extreme wetland ecosystems. The National Wetland Park in the Jimunai alpine periglacial zone, Xinjiang, China, is located within the the Mus Island glacier region and harbours rich plant diversity shaped by its unique geography and environment. In this study, we present an updated checklist of vascular plant of the Park based on a comprehensive literature review, including specimen analysis, database retrieval and field surveys. The revised checklist includes 372 species, 179 genera and 46 families, reflecting a significant increase of 158 species, 45 genera and two families compared to previous records. Notably, the updated list identifies 20 rare and endangered species, four endemic plants of Xinjiang and 23 species newly recorded for the local area. These findings enhance the floristic knowledge of the Jimunai alpine periglacial zone and highlight previously overlooked plant diversity in this extreme habitat. This updated inventory will serve as an essential foundation for further biodiversity research and will be instrumental in guiding effective conservation efforts for this distinct alpine periglacial wetland.

**New information:**

The revised checklist includes 372 species, 179 genera and 46 families, reflecting a significant increase of 158 species, 45 genera and two families compared to previous records. Notably, the updated list identifies 20 rare and endangered species, five endemic plants of Xinjiang and 23 species newly recorded for the local area. These findings enhance the floristic knowledge of the Jimunai alpine periglacial zone and highlight previously overlooked plant diversity in this extreme habitat.

## Introduction

Wetlands, formed by the interaction of land and water, playing a vital role in human survival, development and the maintenance of global biodiversity (Durigan et al. 2022, Chakraborty et al. 2023). Wetlands are collectively recognised with forests and oceans as one of the three major global ecosystems, distinguished by their highly dynamic processes and exceptional biodiversity (Ye 2006, Ding 2020). Due to their ecological significance and resource potential, wetlands are often called "cradle of humanity", "kidneys of the Earth", "biological gene banks", "freshwater sources" and "climate regulators" (Mitsch and Gosselink 2011, Li et al. 2021, Ghimire and Regmi 2024, Yang et al. 2025). Periglacial wetlands, a distinct wetland type found in periglacial regions, represent some of the extreme habitats within wetland ecosystems. They play a crucial role in protecting permafrost, conserving soil and water and sustaining ecosystem stability (Ji 1996, Huang 2020). Within these systems, Wetland plants are fundamental structural components and sensitive bioindicators, reflecting ecological conditions and characterising the ecosystem's fundamental properties and functions (Cronk and Fennessy 2009, Shan et al. 2020, Li et al. 2022). Consequently, studying plant diversity is essential for assessing wetland health, habitat quality and ecosystem stability, providing a critical scientific basis for conservation and sustainable management of wetland biodiversity. Current studies in alpine glacial margin zones primarily focuses on plant origins and phylogeography (Li et al. 1981, Luo 2015), diversity evolution and reproductive strategies (Peng 2015, Ding and Xing 2023), adaptive and functional traits (Xu et al. 2014, Yang et al. 2019, Xing et al. 2024) and species responses to climate change (Dai 2025, Song et al. 2025). However, comprehensive floristic inventories, essential as foundational data for these specialised studies, are often lacking for these extreme environments.

Alpine periglacial wetlands in China are limited in distribution, mainly occurring on the Qinghai-Tibet Plateau, Tianshan, Altai and Qilian Mountains, as well as the Great and Lesser Khingan Mountains and Changbai Mountain (Ji 1996). Only two sites in China are designated as National Wetland Parks at the alpine periglacial level, including Baqing Yuexiongcuo Alpine Periglacial National Wetland Park in Tibet and Jimunai Alpine Periglacial National Wetland Park in Xinjiang. The Jimunai Park is situated on the the northern foothills of the Saur Mountains, encompassing the Mus Island Glacier and its periglacial zone. It represents a quintessential example of temperate inland alpine-subalpine glacial river source wetland within China's arid regions (Mu et al. 2024). Strategically situated in the transitional zone between the Altai and Tianshan mountain ranges, the Park serves as a floristic exchange corridor, facilitating the convergence of plant species from both regions. Its extensive alpine meadows and valley marsh wetlands preserve the pristine ecological characteristics of China's cold temperate regions. The Park’s diverse microhabitats, distinct hydrology and unique geographical conditions create an ideal environment for wild plant survival and reproduction, establishing it as a significant biodiversity hotspot and vital natural gene pool (Zhang et al. 2024). Therefore, the Park serve as a natural laboratory for investigating the impacts of environmental change on temperate alpine-subalpine biodiversity, offering valuable insights for conservation science and ecological research.

Previous research in the Saur Mountains has primarily addressed geological history (Zhou et al. 1999, Zhou et al. 2012), glacial evolution (Zhou et al. 2008, Yu et al. 2022, Mu et al. 2024) and mineral resources (Wu and Han 2024). In the broader Jimunai Region, studies have mostly examined anthropogenically disturbed sites such as ports and reservoirs, reporting on alien plants and phytoplankton (Wang 1984, Yin 1986, Huang et al. 1999, Wu et al. 2010, Wu et al. 2011, Jiang et al. 2020). However, comprehensive research on plant diversity within the Jimunai Alpine Periglacial National Wetland Park remains limited. The only available records consists of an unpublished 2013 appendix to the Park’s Master Plan, documenting 65 species from 45 genera across 19 families. In 2023, Liu Maoxiu provided a broad flora overview, identifying 204 species from 131 genera across 44 families, but without a detailed checklist (Liu et al. 2023). A detailed plant species checklist is a fundamental prerequisite for understanding the flora of any natural geographic region, serving as a foundation for biodiversity conservation, scientific research and sustainable management. To address this critical research gap, this study compiles a comprehensive vascular plant checklist for the Jimunai Alpine Periglacial National Wetland Park, based on an extensive literature review, specimen analysis and nearly three years of field surveys conducted by the Xinjiang Institute of Ecology and Geography's specimen collection team. Our findings provide a scientific basis for local plant conservation and sustainable development strategies, while contributing fundamental data for research on alpine periglacial flora in China.

## Materials and methods

### Overview of the stdudy area

**Figure 1.**
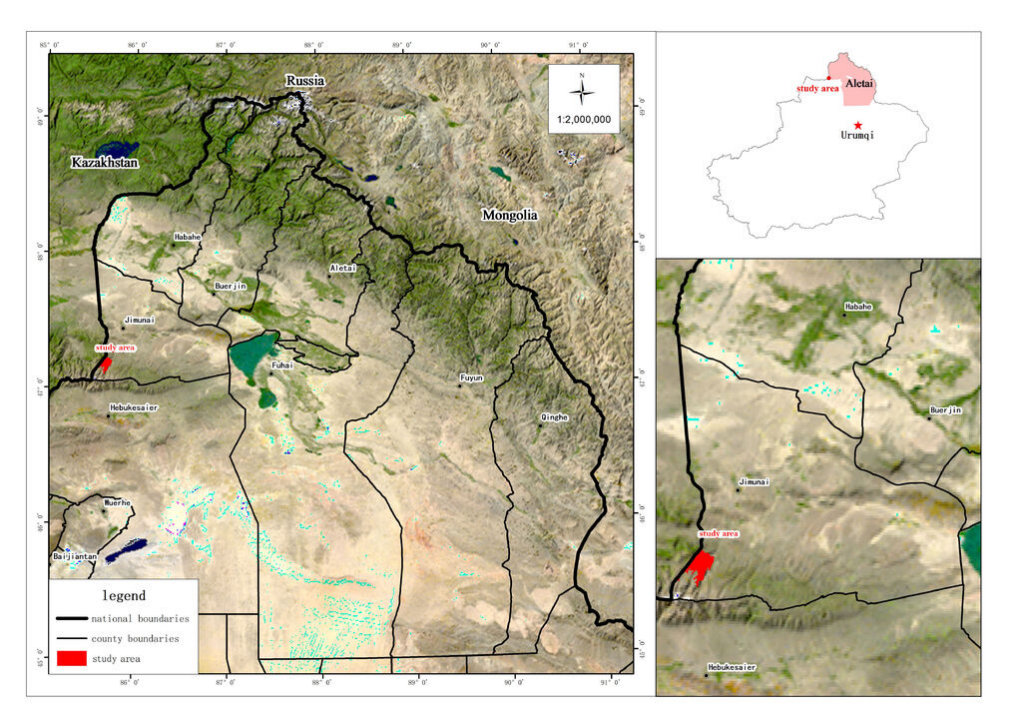
Location map of the study area, the Jimunai Alpine Periglacial National Wetland Park, Xinjiang, China.

**Figure 2.**
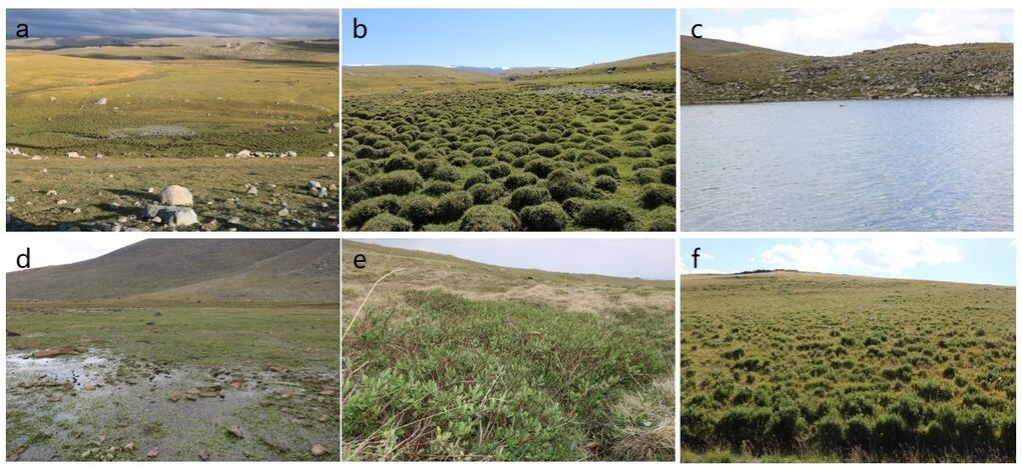
Vegetation landscape and ecological microenvironment of the study area in the Jimunai Alpine Periglacial National Wetland Park. **a** Wetland park ecological landscape; **b** *Carex* peatland landscape; **c** Alpine moraine lake landscape; **d** *Ranunculus natans* C.A.Mey.; **e** Alpine shrub willow shrub; **f** *Dasiphora fruticosa* shrub.

The Jimunai Alpine Periglacial National Wetland Park is situated in south-western Jimunai County, occupying the subalpine and alpine zones of the Saur Mountains. Its geographical coordinates range from 47°6′4″ to 47°10′30″ latitude and 85°33′58″ to 85°42′28″ longitude (Fig. 1). The Park spans approximately 13 km in both the east-west and north-south directions, covering an area of 4,965 hectares. Its elevation ranges from 2,500 to 3,200 metres, with a relative height difference of approximately 700 metres. The Park experiences a temperate continental climate, characterised by distinct seasonal variations, low precipitation, high evaporation and overall arid conditions. It receives ample sunlight, has a short frost-free period and is prone to extreme weather events. Climatic data indicates an average annual temperature of -5.65°C, with 202.2 millimetres of annual precipitation and 4,448.9 hours of annual sunlight. The predominant soil types are meadow soil and alpine permafrost soil, while the zonal vegetation consists mainly of alpine meadows, subalpine meadows and sparse alpine cushion vegetation. The landscape and microenvironment maps of the study area are presented in Fig. 2.

### Plant List Determination

The plant checklist is based primarily on systematic field surveys conducted from May to July each year between 2020 and 2023. During this period, a total of 1,583 voucher specimens were collected and these specimens are preserved at the Herbarium of the Xinjiang Institute of Ecology and Geography, Chinese Academy of Sciences. The final checklist was compiled by integrating our field survey results with data from the Chinese Virtual Herbarium (CVH, https://www.cvh.ac.cn/), National Specimen Resource Sharing Platform (NSII, http://www.nsii.org.cn/), Herbarium of the Xinjiang Institute of Ecology and Geography (XJBI), Chinese Plant Image Database (PPBC, https://ppbc.iplant.cn/), Global Biodiversity Information Facility (GBIF, https://www.gbif-uat.org/) and relevant, newly-published taxonomic articles.

Botanical names were verified using the Taxonomic Name Resolution Service (TNRS, https://tnrs.biendata.org/) and standardised according to Plants of the World Online | Kew Science (POWO, http://powo.science.kew.org/). The taxonomic classification follows the PPG I system (PPG I 2016) for ferns, the Christenhusz system for gymnosperms and the APG IV system (APG IV 2016) for angiosperms.

### Identification of Rare and Endangered Plant Species

The identification of rare and endangered plant species in the Jimunai Alpine Periglacial National Wetland Park, Xinjiang, was determined through multiple authoritative sources. These include the "List of National Key Protected Wild Plants (2021)" (https://www.gov.cn/zhengce/zhengceku/2021-09/09/content_5636409.htm), "List of Key Protected Wild Plants in the Xinjiang Uygur Autonomous Region (2023)" (https://lcj.xinjiang.gov.cn/lcj/xxgkml/202401/5e4bf99f78fd4ba283367510eb6806ff.shtml) and "List of Threatened Species of China's Higher Plants" (Qin et al. 2017). This assessment was further supplemented by global conservation references, such as the "Convention on International Trade in Endangered Species of Wild Fauna and Flora (CITES) 2023" and "IUCN Red List of Threatened Species (2023)" (https://www.iucnredlist.org/). Based on these consolidated criteria, species were assigned a conservation status of critically endangered (CR), endangered (EN) or vulnerable (VU).

### Endemic Plant Identification

The identification of endemic plants in the Jimunai Alpine Periglacial National Wetland Park was based on authoritative regional and national references, including "Endemic Species and Their Distribution in Xinjiang" (Feng et al. 2003), iplant (http://www.iplant.cn/) and "Flora of China".

## Data resources

### Taxonomic checklist

The present contribution builds on the taxonomic inventory of wild vascular plants in the Jimunai Alpine Periglacial National Wetland Park, which is available as a dynamic checklist dataset in GBIF (Cao et al. 2025a).

### Occurrence dataset

The primary data for the distributional records published in the present contribution have been databased in the DarwinCore format and incorporated in the occurrence dataset of wild vascular plants in the Jimunai Alpine Periglacial National Wetland Park, which is available through GBIF (Cao et al. 2025b).

## Checklists

### The checklist of wild vascular plants in the Jimunai Alpine Periglacial National Wetland Park

#### Plantae

D002D6D7-C8AE-5E29-A0D6-C0808F22E92C

https://doi.org/10.15468/bt477z

## Analysis

### Analysis of plant taxonomic composition

The updated inventory for the Jimunai Alpine Periglacial National Wetland Park documents a total of 372 species of wild vascular plants, belonging to 179 genera and 46 families. This represents a substantial increase to previous records, with 28 new families, 147 new genera and 333 new species compared to the 2013 plant catalogue and an increase of three families, 55 genera and 172 species since the 2023 overview. Notably, lycopods and ferns have risen by one family, one genus and one species, while angiosperms have expanded by one family, 44 genera and 157 species. The existing flora is composed of lycopods and ferns (2 families, 2 genera, 3 species), gymnosperms (1 family, 1 genus, 1 species) and angiosperms (43 families, 176 genera, 368 species). Angiosperms dominate the flora, accounting for 93.48% of the families, 98.32% of the genera and 98.92% of all species. Within the angiosperms, dicotyledons are the predominant group, comprising 80.65% of the total plant species recorded in study area (Table 1).

### Analysis of family composition

The composition and dominance of plant families shape the characteristics of the regional flora. A comparison with previous studies reveals significant shifts in the ranking, composition and species diversity of dominant families in the study area (Table 2). In 2013, the dominant families of Cyperaceae, Elaeagnaceae and Amaranthaceae families contained fewer recorded species and later surveys did not classify them as dominant families. The exclusion of Elaeagnaceae and Amaranthaceae, which are representative of Xinjiang's desert flora, underscores the distinct montane and alpine nature of the study area. This indicates that incomplete survey data from 2013 led to inaccuracies in identifying dominant families, emphasising the need for comprehensive plant surveys. The updated 2023 plant catalogue aligns more closely with our updated plant list, though variations exist in the ranking and species numbers within the dominant families. For instance, the Rosaceae family has expanded from 17 to 33 species, shifting its ranking from first to fourth amongst dominant families. The ten dominant families in the Jimunai Alpine Periglacial National Wetland Park are Poaceae, Ranunculaceae, Asteraceae, Rosaceae, Fabaceae, Brassicaceae, Gentianaceae, Lamiaceae, Caryophyllaceae and Polygonaceae. Together, these families encompass 112 genera and 245 species, accounting for 62.57% of the total genera and 65.86% of the total species in the region. This clearly demonstrates a pronounced dominance phenomenon, where plant species are concentrated within a few large families.

### Analysis of genus composition

Genera exhibit relatively stable taxonomic and ecological characteristics with pronounced regional variations, making them more reflective of local plant traits than families. A comparative analysis of dominant genera across the 2013, 2023 and 2024 inventories reveals three consistently dominant genera: *Carex*, *Ranunculus* and *Thalictrum*. However, differences exist in the composition, ranking and species count within dominant genera over time (Table 3). In 2013, the inventory recorded 45 genera, but apart from the three shared dominant genera, others such as *Persicaria*, *Lepidium*, *Primula*, *Leontopodium* and *Taraxacum* contained only two species each. As subsequent, more comprehensive surveys recorded greater diversity, the relative dominance of these genera diminished. The 2023 and 2024 inventories share five dominant genera, though their rankings shifted with increasing species counts. For example, *Ranunculus* rose from seventh to first as its species count increased from three to seven, while *Gentiana* dropped from first to second, despite its species count doubling from five to ten species. Ultimately, the top ten dominant genera of seed plants in the Jimunai Alpine Periglacial National Wetland Park are *Ranunculus*, *Gentiana*, *Allium*, *Carex*, *Potentilla*, *Festuca*, *Poa*, *Thalictrum*, *Rhodiola* and *Oxytropis*. These genera are characteristics of high-altitude regions in Xinjiang, a distribution that aligns with the alpine periglacial environment of the Wetland Park.

### Analysis of the life forms of wild vascular plants in the Jimunai Alpine Periglacial National Wetland Park

**Figure 3.**
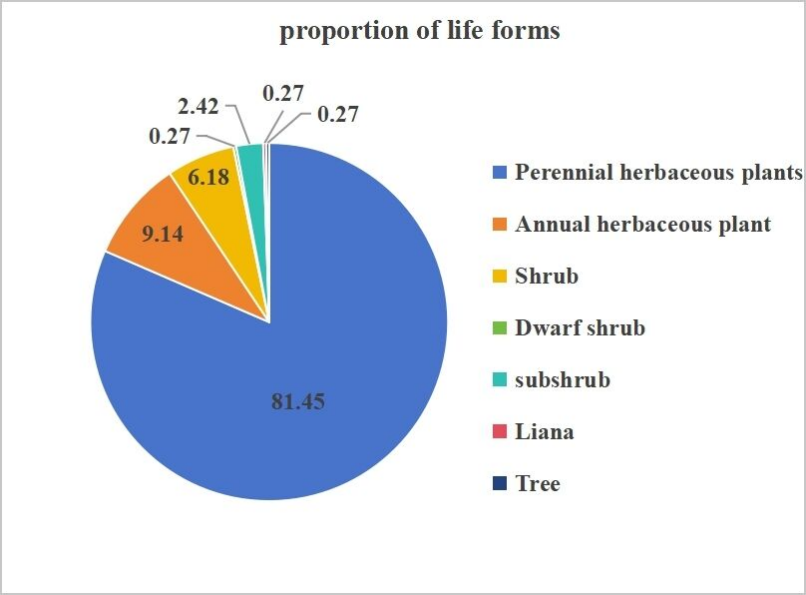
Life forms of wild vascular plants in the Jimunai Alpine Periglacial National Wetland Park.

The wild vascular plants of the Jimunai Alpine Periglacial National Wetland Park were categorised into seven life forms, based on life history and degree of lignification including trees, shrubs, lianas, semi-shrubs, small semi-shrubs, perennial herbs and annual herbs (Fig. 3). Herbaceous plants dominate the flora, with a total of 337 species, accounting for 90.59% of the total wild vascular plant species, with perennial herbs being the most prevalent at 81.45%. In contrast, woody plants are scarce, represented by only 33 shrub species (8.87%) and a single tree species (*Larix sibirica*). Lianas are also mimnimally represented, with only one species (0.27%). The shrubs primarily consist of low-growing, cold-resistant species such as *Dasiphora fruticosa*, *Salix arctica*, *Salix vestita* and *Berberis sibirica*. This life-form spectrum is a direct reflection of the local alpine condition. The high altitude, low temperatures and extended winter season create harsh conditions for woody plants development. Simultaneously, the short growing season and low effective accumulated temperature further restrict the growth and reproduction of annual plants. Consequently, perennial herb dominate, as their growth form is best adapted to survive the harsh winters and complete their life cycle during short summer in this cold alpine environment of the Wetland Park.

### Analysis of rare, endangered and key protected plant species

**Figure 4.**
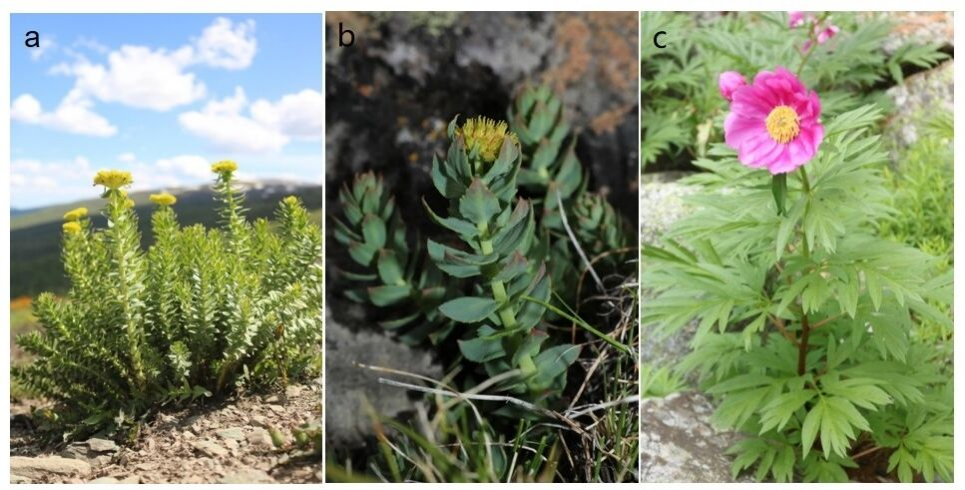
A few glimpses of are and endangered protected plants. **a** *Rhodiola rosea* L.; **b** *Rhodiola heterodonta* (Hook.f. & Thomson) Boriss.; **c** *Paeonia anomala* L.

The Jimunai Alpine Periglacial National Wetland Park harbours a rich diversity of rare and endangered plant species (Table 4), comprising 20 species across 10 genera and 11 families, accounting for 5.38% of the total documented flora. Amongst them, *Allium altaicum* is classified as Near Threatened (NT) by the International Union for Conservation of Nature (IUCN) Red List (2023). The List of Threatened Species of China's Higher Plants (2017) includes 13 species, with one categorised as Endangered (EN), seven as Near Threatened (NT) and five as Vulnerable (VU). Furthermore, three species, *Tulipa altaica*, *Rhodiola rosea*, and *Rhodiola quadrifida*, are listed as The National Key Protected Plants (2021) (Level II). Additionally, the Xinjiang Key Protected Plants (2023) identifies nine species, including three Level I protected species (Lilium martagon var. pilosiusculum, *Paeonia anomala* and *Paeonia intermedia*) and six Level II species (*Arctous alpina*, *Allium altaicum*, *Rhodiola coccinea*, *Rhodiola gelida*, *Rhodiola heterodonta* and *Rhodiola litwinovii*). The Park is also home to four endemic species of Xinjiang including *Elymus glaberrimus*, *Ranunculus chinghoensis*, *Draba fuhaiensis* and *Linaria longicalcarata*. These endemics are of considerable scientific value for understanding the origin, differentiation and evolution of regional flora. Representative rare, endangered and endemic species are shown in Fig. 4.

### Analysis of newly-recorded species in local distribution

**Figure 5.**
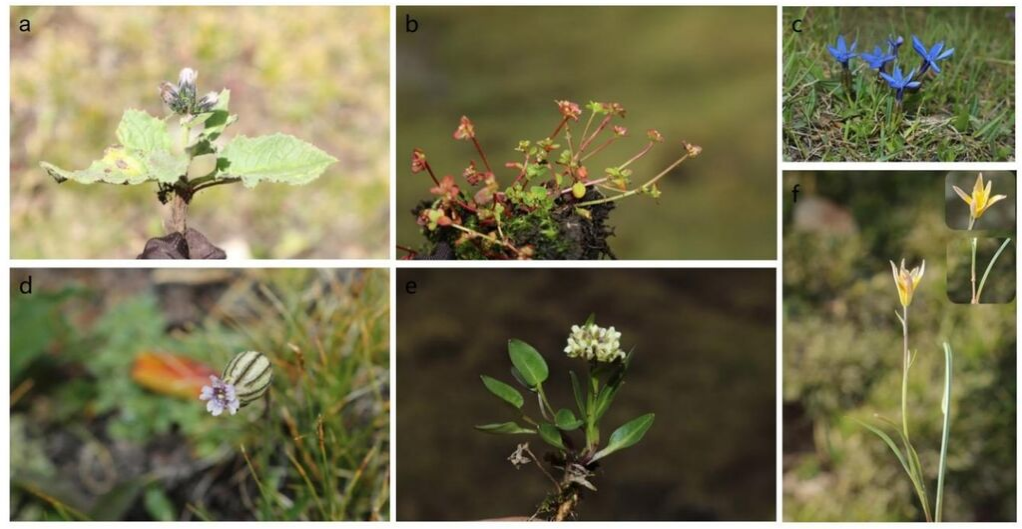
Images of newly-recorded species in local distribution. **a** *Saussurea elliptica* C.B.Clarke ex Hook.f.; **b** *Koenigia islandica* L.; **c** *Gentiana verna* L.; **d** *Silene karaczukuri* B. Fedtsch.; **e** *Eutrema fontanum* (Maxim.) Al-Shehbaz & Warwick; **f** *Gagea jaeschkei* Pascher.

The statistical analysis of species distribution data revealed 23 newly-recorded vascular plant species in Jimunai County and Saur Mountains (Table 5, Suppl. material 1). These findings provide valuable insights into the geographical composition, floristic characteristics and biodiversity of the local flora, while confirming the study area’s role as a convergence zone for plant species from the Altai, Tianshan and Balluk Mountains. For example, alpine willows, such as *Salix arctica*, *S. vestita* and *S. berberifolia*, were previously recorded only in the gravel zones of the Altai Mountains, while *Ranunculus alberti* and *Anemonastrum protractum* were known from the Tianshan Mountains. Additionally, species like *Rhodiola heterodonta*, *Gagea jaeschkei* and *Saussurea elliptica* were documented in Wuqia and Tashkurgan Counties on the Pamir Plateau, situated at the junction of the Tianshan and Kunlun Mountains. Notably, *Silene karaczukuri* was previously recorded only in Yecheng County, deep within the Kunlun Mountains. The presence of these species in the Saur Mountains highlights the habitat diversity and unique alpine periglacial ecosystem, further emphasising the need for continued biodiversity research and conservation. Some of these newly-recorded species are illustrated in Fig. 5 .

## Discussion

Over the past decade, plant diversity in Jimunai Alpine Periglacial National Wetland Park has increased significantly. The Park now holds 372 species of wild vascular plants from 46 families and 179 genera. The flora constitutes 65.71%, 57.28% and 53.76% of the families, genera and species respectively, recorded for the Chinese portion of the Saur Mountains (Han 1984), establishing the Park as an important centre of plant dicersity within this mountain range. Notably, 158 species across 45 genera and two families are newly recorded for the Park. The rate of species accumulation in Jimunai has been significantly faster than in Tianshan Glacier No. 1, where 66 species were recorded in 1998, 74 in 2000 and only 50 in 2012 (Wei et al. 1998, An et al. 2000, Liu et al. 2012, Ding and Xing 2023). The decline at Tianshan Glacier No. 1 underscores the environmental heterogeneity of different alpine periglacial zones and the uniqueness of their ecological processes. Several interconnected factors likely contribute to the observed floristic increase in Jimunai. Initially, before its designation as a National Wetland Park, the area was situated on the fringes of alpine ice, characterised by a harsh climate and limited accessibility, resulting in low botanical research and documentation. Additionally, grazing was the primary livelihood of local residents and human disturbances, such as overgrazing, impacted the ecological balance, reducing plant abundance. The transformation of Jimunai into a National Wetland Park, alongside the protection of the Musi Island Glacier, has significantly mitigated human disturbances, fostering a more stable environment for plant growth. The updated plant inventory can reflect the improvement of the plant species richness in the Jimunai Alpine Periglacial National Wetland Park. Another critical factor is global warming, which has led to melting ice and snow, increasing water availability across the Wetland Park, thus creating favourable conditions for vegetation expansion. Furthermore, climate change is driving a northwards shift in climatic zones, causing many species and vegetation types to extend their distribution to higher latitudes (Zhao et al. 2023). This is evidenced by the presence of desert plants, such as *Krascheninnikovia ceratoides*, *Eremopyrum triticeum* and *Ancathia igniari*, which were previously uncommon in the region, highlighting the evolving ecological dynamics of the Wetland Park.

The life forms of plants in Jimunai Wetland Park is influenced by various ecological factors, such as altitude, temperature, topography, soil and light (Zheng et al. 2024). The plateau environment, characterized by fluctuating climate, rugged terrain and gravelly soil conditions, limits the presence of woody plants, favouring the growth of low, cold-resistant species, such as alpine willows and *Dasiphora fruticosa*. Additionally, the Park's location near the Musi Island Glacier in Saur Mountains results in glacial meltwater feeding multiple streams that spread across the wetland. These streams create perennial and seasonal water depressions, forming a unique microclimate that supports the growth of cold-resistant and hygrophilous herbaceous plants. As a result, the dominant vegetation in the Park consists of alpine and subalpine meadows, shaped by the area's climatic and hydrological conditions. The prevalence of perennial herbs, which can delay reproduction until conditions are favourable, are especially represented in alpine floras (Li 2023). This characteristic is evident in Jimunai, where perennials account for 81.19% of species, closely aligning with Sanjiangyuan National Park (78.73%) and consistent with life-form composition in similar alpine environments (Zhang et al. 2019, Sun 2023).

Jimunai Alpine Periglacial National Wetland Park serves as a critical sanctuary of rare, endangered and endemic species, with 22 rare and endangered species recorded in the current checklist. Its unique geographical location at the northern foot of the Saur Mountains, where the floras of the Altai Mountains, Tianshan Mountains, Barluk Mountains and Junggar Desert converge, creates a natural corridor for plant exchange, hybridisation and specialisation (Xinjiang Synthetical Investigation Team of the Chinese Academy of Sciences, Institute of Botany of the Chinese Academy of Sciences 1978). The Park’s high, isolated mountains function as biological islands, fragmenting plant distributions and fostering unique, localised flora. This process is further amplified by the diverse microenvironments shaped by altitude, moisture and soil variations further support rare and endemic species (Yang et al. 2021). As a transboundary protected area adjacent to the Jimunai Alpine Periglacial National Wetland Park, Katon-Karagaysky National Park has 30 species of protected plants listed in the Red Book of the Republic of Kazakhstan (Vaganov et al. 2023) , which is eight more than that in the study area. Amongst the protected plants in both areas, *Rhodiola rosea* L. and *Paeonia anomala* L. are the shared representative species. These species can not only well adapt to the cold and arid habitat at high altitudes, but also are characteristic groups of the Central Asian mountain flora. Their transboundary distribution not only reflects the historical connectivity of the flora between the two areas, but also confirms the supporting role of similar ecological environments for the survival of species. Therefore, we call for strengthening the cooperation on the joint protection of biodiversity in transboundary protected areas between China and neighbouring countries, to jointly build a community with a shared future for all life on Earth (Duan et al. 2025).

The newly-updated plant checklist provides fundamental baseline data for studying plant diversity in Jimunai Wetland Park. The substantial increase in recorded species highlights both the previous insufficiency of plant diversity surveys in the alpine periglacial zone and the positive impact of protected area establishment on local flora conservation. The region’s harsh climate, complex terrain and island-like biological barriers contribute to the difficulty of field investigations, compounded by the short growing season, microenvironmental variations and phenological disparities amongst species (Xu and Chen 2020). Survey timing, investigator expertise and sampling locations also influence recorded species composition. Upon reviewing past data, species such as *Halostachys caspica*, *Halocnemum strobilaceum* and *Nitraria sphaerocarpa*, which are typical desert saline-alkali plants, were removed from the species list, as no supporting specimens or suitable habitats were found in the Reserve. Additionally, the presence of *Primula sinensis* in the study area remains unverified, warranting further investigation. Given the gaps in plant diversity research, we advocate for more detailed and ongoing biodiversity surveys in Xinjiang’s alpine periglacial zones. In addition, the enviromental heterogeneity and geographical isolation of "sky islands" in alpine periglacial zones make their species diversity and bio-ecological processes unique and irreplaceable (Shen et al. 2017). Comparative studies of species diversity across different mountainous areas and within regions of the same mountain range are crucial for understanding biodiversity formation and maintenance, as well as species responses to climate change (Chen et al. 2011, Zu and Wang 2022). We, therefore, recommend that future research focus on comparing plant compositions in the alpine periglacial zones of the study area with adjacent transboundary protected areas, especially in Katon-Karagaysky and Tarbagatay National Parks, to provide background data on vegetation dynamics and climate change responses in Central Asia.

## Supplementary Material

XML Treatment for Plantae

FA8C94BA-6D9C-5B18-89F0-6EB809EB99FE 10.3897/BDJ.13.e160471.suppl1 Supplementary material 1 Occurrence data Data type xls Brief description Occurrence of newly-recorded vascular plant species in Jimunai County and Saur Mountains. File: oo_1466944.xls https://binary.pensoft.net/file/1466944 Qiumei Cao

## Figures and Tables

**Table 1.** Composition of Wild Vascular Plants in the Jimunai Alpine Periglacial National Wetland Park across different taxonomic reports.

**List source**	**2013 Master Plan List**				**The plant list surveyed by Liu Maoxiu in 2023**				**Current study**			
Taxa	families	genera	species	Percentage（%）	families	genera	species	Percentage（%）	families	genera	species	Percentage（%）
Lycophytes and ferns	0	0	0	0.00	1	1	2	0.98	2	2	3	0.81
Gymnosperm	0	0	0	0.00	1	1	1	0.49	1	1	1	0.27
Angiosperm	19	45	65	100.00	42	129	201	98.53	43	176	368	98.92
Monocotyledon	2	8	18	27.69	5	15	20	9.80	5	29	68	18.28
Dicotyledon	17	37	47	72.31	37	114	181	88.73	38	147	300	80.65
Total	19	45	65	100.00	44	131	204	100.00	46	179	372	100.00

**Table 2.** Statistics of the variation in genera and species of dominant seed plants in the Jimunai Alpine Permafrost National Wetland Park.

**2013 Master Plan List**			**2023 List of Plants**			**Current study**		
Families	Genera	Species	Families	Genera	Species	Families	Genera	Species
Asteraceae	7	9	Rosaceae	10	17	Poaceae	22	43
Poaceae	5	9	Ranunculaceae	8	16	Ranunculaceae	10	34
Cyperaceae	4	9	Fabaceae	9	13	Asteraceae	20	34
Brassicaceae	3	7	Asteraceae	7	13	Rosaceae	13	33
Polygonaceae	4	5	Gentianaceae	6	12	Fabaceae	11	23
Ranunculaceae	3	5	Lamiaceae	6	11	Brassicaceae	10	20
Rosaceae	4	4	Brassicaceae	7	10	Gentianaceae	6	17
Fabaceae	3	3	Poaceae	10	10	Lamiaceae	7	16
Amaranthaceae	2	2	Polygonaceae	6	9	Caryophyllaceae	6	14
Nitrariaceae	2	2	Crassulaceae	4	7	Polygonaceae	7	11

**Table 3.** Statistics of dominant genera of seed plants in the Jimunai Alpine Periglacial National Wetland Park.

**S erial** **number**	**Composition of Dominant** **Genera in 2013**		**Composition of Dominant** **Genera in 2023**		**Composition of Dominant** **Genera in 2024**	
	Genus	No. of species	Genus	No. of species	Genus	No. of species
1	* Carex *	6	* Gentiana *	5	* Ranunculus *	14
2	* Draba *	4	* Dracocephalum *	5	* Gentiana *	10
3	* Poa *	4	* Thalictrum *	4	* Allium *	9
4	* Persicaria *	2	* Saxifraga *	4	* Carex *	9
5	* Ranunculus *	2	* Rosa *	4	* Potentilla *	9
6	* Thalictrum *	2	* Carex *	4	* Festuca *	7
7	* Lepidium *	2	* Bistorta *	3	* Poa *	7
8	* Primula *	2	* Ranunculus *	3	* Thalictrum *	6
9	* Artemisia *	2	* Draba *	3	* Rhodiola *	6
10	* Taraxacum *	2	* Rhodiola *	3	* Oxytropis *	6

**Table 4.** List of rare and endangered, key protected and endemic plant species.

**Latin name**	**Rank of** **states**	**Xinjiang** **Level**	**Rare and** **endangered** **species**	**IUCN**	**CITES**	**Endemic Plants** **of Xinjiang**
*Larix sibirica* Ledeb.			VU			
Lilium martagon var. pilosiusculum Freyn		Ⅰ	NT			
*Tulipa altaica* Pall. ex Spreng.	Ⅱ					
*Allium altaicum* Pall.		Ⅱ	NT	NT		
*Allium hymenorhizum* Ledeb.			NT			
*Allium setifolium* Schrenk ex Fisch. & C.A.Mey			NT			
*Elymus glaberrimus* (Keng & S. L. Chen) S. L. Chen						＋
*Ranunculus altaicus* Laxm.			NT			
*Ranunculus chinghoensis* L.Liu			VU			＋
*Paeonia anomala* L.		Ⅰ	VU			
*Paeonia intermedia* C.A.Mey.		Ⅰ	VU			
*Rhodiola coccinea* (Royle) Boriss.		Ⅱ			Ⅱ	
*Rhodiola gelida* Schrenk ex Fisch. & C.A.Mey		Ⅱ			Ⅱ	
*Rhodiola heterodonta* (Hook. f. & Thomson) Boriss.		Ⅱ			Ⅱ	
*Rhodiola litwinovii* Boriss.		Ⅱ			Ⅱ	
*Rhodiola quadrifida* (Pall.) Schrenk, in Fisch. & C. A. Mey.	Ⅱ				Ⅱ	
*Rhodiola rosea* L.	Ⅱ		VU		Ⅱ	
*Salix berberifolia* Pall.			NT			
*Salix pyrolifolia* Ledeb.						
*Salix vestita* Pursh			EN			
*Chorispora greigii* Regel			NT			
*Draba fuhaiensis* Z.X.An						＋
*Arctous alpina* (L.) Nied.		Ⅱ				
*Linaria longicalcarata* D. Y. Hong						＋

**Table 5.** New distribution of local records.

Latin name	New distribution of local records
*Salix arctica* Pall.	＋
*Salix berberifolia* Pall.	＋
*Salix vestita* Pursh	＋
*Koenigia islandica* L.	＋
*Bistorta elliptica* (Willd. ex Spreng.) V.V.Petrovsky, D.F.Murray & Elven	＋
*Silene karaczukuri* B.Fedtsch.	＋
*Cerastium elongatum* Pursh	＋
*Ranunculus alberti* Regel & Schmalh.	＋
Ranunculus pulchellus var. pulchellus	＋
*Anemonastrum protractum* (Ulbr.) Holub	＋
*Draba fuhaiensis* Z.X.An	＋
*Draba sachalinensis* (F.Schmidt) Trautv.	＋
*Eutrema altaicum* (C.A.Mey.) Al-Shehbaz & Warwick	＋
*Eutrema fontanum* (Maxim.) Al-Shehbaz & Warwick	＋
*Rhodiola heterodonta* (Hook. f. & Thomson) Boriss.	＋
*Rhodiola gelida* Schrenk ex Fisch. & C.A.Mey.	＋
*Rhodiola coccinea* (Royle) Boriss.	＋
*Potentilla evestita* Th. Wolf	＋
*Potentilla crantzii* (Crantz) Beck ex Fritsch	＋
*Gentiana verna* L.	＋
*Saussurea elliptica* C. B. Clarke ex Hook.f.	＋
*Galatella fastigiiformis* Novopokr.	＋
*Gagea jaeschkei* Pascher	＋
